# The Mental Health Recovery Measure Can Be Used to Assess Aspects of Both Customer-Based and Service-Based Recovery in the Context of Severe Mental Illness

**DOI:** 10.3389/fpsyg.2016.01679

**Published:** 2016-11-03

**Authors:** Albino J. Oliveira-Maia, Carina Mendonça, Maria J. Pessoa, Marta Camacho, Joaquim Gago

**Affiliations:** ^1^Champalimaud Clinical Centre, Champalimaud Centre for the UnknownLisboa, Portugal; ^2^Champalimaud Research, Champalimaud Centre for the UnknownLisboa, Portugal; ^3^Department of Psychiatry and Mental Health, Centro Hospitalar de Lisboa OcidentalLisboa, Portugal; ^4^Department of Psychiatry and Mental Health, NOVA School of Medicine – Faculdade de Ciências Médicas, Universidade Nova de LisboaLisboa, Portugal; ^5^Department of Psychiatry and Mental Health, Centro Hospitalar Cova da BeiraCovilhã, Portugal

**Keywords:** recovery, quality of life, needs assessment, schizophrenia, schizoaffective disorder

## Abstract

Within clinical psychiatry, recovery from severe mental illness (SMI) has classically been defined according to symptoms and function (service-based recovery). However, service-users have argued that recovery should be defined as the process of overcoming mental illness, regaining self-control and establishing a meaningful life (customer-based recovery). Here, we aimed to compare customer-based and service-based recovery and clarify their differential relationship with other constructs, namely needs and quality of life. The study was conducted in 101 patients suffering from SMI, recruited from a rural community mental health setting in Portugal. Customer-based recovery and function-related service-based recovery were assessed, respectively, using a shortened version of the Mental Health Recovery Measure (MHRM-20) and the Global Assessment of Functioning score. The Camberwell Assessment of Need scale was used to objectively assess needs, while subjective quality of life was measured with the TL-30s scale. Using multiple linear regression models, we found that the Global Assessment of Functioning score was incrementally predictive of the MHRM-20 score, when added to a model including only clinical and demographic factors, and that this model was further incremented by the score for quality of life. However, in an alternate model using the Global Assessment of Functioning score as the dependent variable, while the MHRM-20 score contributed significantly to the model when added to clinical and demographic factors, the model was not incremented by the score for quality of life. These results suggest that, while a more global concept of recovery from SMI may be assessed using measures for service-based and customer-based recovery, the latter, namely the MHRM-20, also provides information about subjective well-being. Pending confirmation of these findings in other populations, this instrument could thus be useful for comprehensive assessment of recovery and subjective well-being in patients suffering from SMI.

## Introduction

Recovery is a concept that cuts across medicine, with particular importance in the context of chronic disease. Clinical definitions of recovery are generally related to reduction or remission of symptoms and return to pre-morbid or full levels of functioning. However, these definitions are variable according to disease or disorder and, frequently, consensus definitions are difficult to obtain. In the contexts of clinical psychiatry, mental health policy and psychiatric research, the concept of recovery from severe mental illness (SMI) has become increasingly relevant (Slade, 2010). This is particularly true for conditions such as schizophrenia, where recovery is very heterogeneous (Lieberman et al., 2008), since there are arguments that stigma and negative stereotyping are self-fulfilling attitudes directly resulting from a misconception of a very limited potential for recovery (Liberman and Kopelowicz, 2005).

The conceptualization of recovery has been challenging for psychiatric disorders, in part because different groups use the term differently. Clinical psychiatry has traditionally defined recovery based on symptoms and several dimensions of function (service-based, objective, or clinical recovery – SBR), while consumer movements advocate for recovery to be defined as the process that involves overcoming mental illness, regaining self-control and establishing a meaningful and fulfilling life (customer-based, subjective, or personal recovery – CBR) (Schrank and Slade, 2007). In patients suffering from schizophrenia, recovery has many predicting factors, including socio-demographic variables, among others (Westermeyer and Harrow, 1984; Wieselgren et al., 1996). Specifically, age and functional status at onset, better cognitive functioning at stabilization, shorter duration of psychosis and early remission seem to best predict functional SBR (Robinson et al., 2004; Lambert et al., 2008). While there is less data for CBR, it has been proposed that subjective well-being at onset and marital satisfaction are associated with increased subjective recovery (Lambert et al., 2008; Tse et al., 2014). In any case, the concept of CBR has gained increasing relevance, given the movements toward promotion of patient-centered medicine and patient engagement in healthcare (Barello et al., 2012; Mullins et al., 2012; Domecq et al., 2014; Richards et al., 2015).

Unfortunately, lack of precision in the definition of these constructs and their dimensions has lead to variable use of these terms within the literature. In fact, CBR has been directly or indirectly equated to other measures of subjective experience, such as quality-of-life (QoL) (Roe et al., 2011), and the degree to which CBR and SBR are separable constructs is not consensual (Resnick et al., 2004; Andresen et al., 2010; Lloyd et al., 2010; Roe et al., 2011, 2012; Norman et al., 2013; Stanhope et al., 2013). To address this question empirically, as we propose here, stringent conceptualizations of these recovery constructs have been considered by several authors. Specifically it has been proposed that the distinction between CBR and SBR should result from the methods according to which the two constructs and their respective measurement instruments are defined and derived (Campbell-Orde et al., 2005; Andresen et al., 2010). According to these conceptualizations, CBR is considered to be recovery defined by users/patients and measured by instruments developed according to the accounts of users/patients (e.g., focus groups, qualitative analysis of patient interviews). SBR, on the other hand, is recovery defined by service providers and experts, and is measured using instruments developed according to the expertise of service providers and experts (Schrank and Slade, 2007). Nevertheless, this approach to distinguish recovery constructs is questionable, and there has been insufficient empirical work to support the distinction between the two.

Here, we set out to clarify the relationship between CBR and SBR constructs in a population of patients with SMI. Since there are no validated instruments for measurement of CBR in Portuguese patients with SMI, we initially translated and validated the Mental Health Recovery Measure (MHRM) (Bullock and Young, 2003; Young and Bullock, 2005) for use in this patient population. This instrument was chosen because it is one of only two self-rated measures of CBR according to the stringent definition presented above (Campbell-Orde et al., 2005; Andresen et al., 2010), i.e., it was developed according to the accounts of service-users. Furthermore, the MHRM has several versions with excellent psychometric properties (Bullock and Young, 2003; Young and Bullock, 2005; Chang et al., 2013; Armstrong et al., 2014) and has been successfully translated and validated into other languages (van Nieuwenhuizen et al., 2014). Once this instrument was validated, we proceeded to compare customer-based and service-based recovery and clarify their differential relationship with other constructs, namely needs and subjective QoL. The four constructs were assessed simultaneously using either clinician-reported (SBR and needs) and/or self-reported (CBR, needs and subjective QoL) measures.

## Materials and Methods

### Participants

A sample of 101 users of a community mental health service (CMHS) at the Department of Psychiatry and Mental Health of Centro Hospitalar Cova da Beira (CHCB) in Covilhã, Portugal, was recruited. The ethics committee of CHCB approved the study and written informed consent was obtained from all participants, in accordance with the declaration of Helsinki. Individuals diagnosed with schizophrenia or schizoaffective disorder according to the 10th revision of the International Classification of Disorders (World Health Organization, 1993 – diagnostic codes F20 and F25, respectively), were eligible for enrolment and were identified by review of the institutional patient database, with diagnosis confirmed by chart review. Eligible individuals were contacted consecutively from January 2010 to December 2011, to schedule data collection. Exclusion criteria included illiteracy, presence of comorbid dementia or development disorder, acute exacerbation of positive psychotic symptoms, acute intoxication with alcohol or other substances or acute non-psychiatric disease. Patients no longer receiving regular clinical care at the CMHS were also excluded.

### Mental Health Recovery Measure

To measure CBR, we used the MHRM, developed by Young and Bullock at the University of Toledo) (Bullock and Young, 2003; Young and Bullock, 2005). The development of this self-report instrument was based on theoretical analysis of qualitative interview data regarding recovery-related experiences of persons with SMI, namely recurrent major depression, bipolar disorder, or schizophrenia spectrum disorders (Young and Ensing, 1999), followed by a series of revisions according to formal psychometric analyses (Young and Bullock, 2005). The current English version of the MHRM includes 30 items (MHRM-30), scored using a five point Likert scale that are added in a total score ranging from 0 (low recovery) to 120 (high recovery). The scale has eight domains: overcoming stuckness, self-empowerment, learning and self-redefinition, basic functioning, overall well being, new potentials, advocacy/enrichment and spirituality. The Flesch–Kincaid reading level is grade 7–8 and the administration time is of approximately 5 min. To develop a Portuguese version of this scale, translation, back-translation and adaptation of the MHRM-30 was performed by a team of three bilingual researchers, with expertise in clinical psychiatry, in order to obtain a final consensus version for application in a Portuguese population. Psychometric refinement of the original scale was performed according to results of an exploratory factor analysis (Young and Bullock, 2005) of MHRM-30 items with adequate item-total correlation, followed by item-reduction for domains with five or more items, following increasing order of item-domain correlations, until effects on Cronbach’s α for that domain were no longer negligible or a minimum number of four items was reached. According to these methods, a smaller 20-item Portuguese version of the MHRM (MHRM-20) was obtained.

### Other Evaluation Instruments

Quality-of-life was assessed using the TL-30S, a shortened version of the Lehman QoL scale which has been used extensively in individuals with SMI (Lehman, 1996). A subjective subscale score was calculated by adding scores from Likert scales for satisfaction in eight life domains (living situation, family, social relations, leisure, work, safety, finances, and physical health), as well as general life satisfaction, each rated from terrible (=1) to delighted (=7) (Dixon et al., 2007). The Camberwell Assessment of Need (CAN) assesses the clinical and social needs of people with SMI over the previous month, across 22 mental health and social domains (Phelan et al., 1995). Each domain is rated on a 3-point scale from the absence of need (=0) to the presence of an unmet need (=2), and a total score is calculated by adding the domain ratings. The CAN may be applied separately according to the perspective of the user, a clinical staff member or a caretaker (Phelan et al., 1995). The Global Assessment Functioning Score (GAF) was used as a clinician-rated measure of function-related service-based recovery. This is a global scale to measure psychological, social and occupational functioning on a hypothetical continuum ranging from 0 (maximal dysfunction from mental illness) to 100 (high functioning and health) (Jones et al., 1995), with evidence for concurrent validity to assess functioning in patients suffering from schizophrenia (Startup et al., 2002). Portuguese translations of psychometric instruments, with extensive prior use in patients suffering from SMI (Gago, 1996; Fernandes et al., 2009; Brissos et al., 2012; Talina et al., 2013; Cardoso et al., 2016), were used.

### Data Collection

A mental health nurse and a psychiatrist collected demographic, clinical and psychometric data for each patient on a single occasion. One-hundred-and-one individuals were recruited and evaluations were performed when users visited the outpatient clinic of the CMHS, or when the community mental health team visited their home. One team member oversaw the self-administration of the MHRM-30 and TL-30S scales while the alternate team member, thus blinded to the MHRM-30 and TL-30S responses, interviewed the participant to obtain clinical data, apply the CAN scale and assess the GAF score. In a convenience subsample of 40 participants, the MHRM-30 was applied again approximately 3 months later, to assess test-retest reliability. Participants in this subsample were slightly younger than the remainder of the sample (48 ± 14.9 vs. 55 ± 12.4 years, *p* < 0.01), but not significantly different regarding education or duration of disease (*p* > 0.05, *t*-tests, data not shown), nor regarding gender, marital status and substance abuse status (*p* > 0.05, χ^2^ tests, data not shown).

### Data Analysis

Data were analyzed using SAS software (version 9.3, SAS Institute, Cary, NC, USA). All continuous measurements were normally distributed according to skewness, kurtosis and comparison of mean and median. Sequential multiple linear regression models were used to test the association between MHRM-20 and GAF scores, when adjusting for other psychometric scores and for demographic variables. In these models, model assumptions were tested by analyses of the distribution of residuals and influence diagnostics were conducted using Cook’s distance. Data transformations and polynomial models were used to test the better alternative to fit continuous predictors. Data for duration of disease were omitted from these models due to concerns about multicollinearity.

## Results

Demographic, clinical and psychometric data are summarized in **Table 1**. None of the patients invited for the study declined to participate. However, six participants did not complete the MHRM-30 scale, three of which also did not complete the TL-30S scale.

**Table 1:** Description of the data collected for this study.

Variable^1^	% or Mean ± SD	Range
Gender (% male)	76.2%	–
Marital status (% married^2^)	27.7%	–
Substance abuse (% positive^3^)	34.7%	–
Psychiatric home care (%)	12.9%	–
Age (years)	52.2 ± 13.8	18–83
Education (years)	6.3 ± 3.8	0–16
Duration of disease (years)	24.4 ± 11.9	1–49
MHRM-30 (total score)	74.8 ± 15.2	43–115
MHRM-20 (total score)	49.4 ± 12.5	20–78
TL30S (subjective subscore)	45.7 ± 7.7	27.5–60
CAN user (total score)	8 ± 4.4	0–20
CAN staff (total score)	9.3 ± 5.4	0–28
GAF (score)	49.6 ± 19.4	10–90

*^1^Number of observations for all variables was 101, with the exception of MHRM (n = 95) and TL30s (n = 98). ^2^Percentage of individuals legally married and not separated, or cohabiting with a primary partner. ^3^Percentage of individuals that self-report abuse of alcohol or illicit substances. Range, Minimum and maximum values; mean ± SD, Mean and standard deviation.*

### Psychometric Properties of the Portuguese Version of the MHRM

Regarding the full Portuguese translation of the MHRM (MHRM-30), while overall internal consistency was high (Cronbach’s α = 0.9), it was medium-low or low for several domains, specifically ‘overcoming stuckness,’ ‘self-empowerment,’ ‘basic functioning,’ and ‘advocacy/enrichment’ (0.36 < α < 0.65). The remaining domains (‘learning and self-redefinition,’ ‘overall well-being,’ ‘new potentials,’ and ‘spirituality’) had adequate internal consistency (0.70 < α < 0.98; Supplementary Table 1). Furthermore, eight items of the full scale had item-total correlations less than 0.40 (Supplementary Table 2). Thus, psychometric refinement of the full scale was performed according to an exploratory factor analysis of the 22 original MHRM items with adequate item-total correlation (Supplementary Table 3). The items were thus assigned to six novel domains, several of which corresponded, at least in part, to the domains in the original scale with higher α, and which were thus similarly named (‘empowerment,’ ‘redefinition,’ ‘identity,’ ‘social functioning,’ ‘overall well-being,’ and ‘optimism’). Item-reduction (see Materials and Methods) resulted in a 20-item revised MHRM scale (MHRM-20), consisting of six domains with adequate internal consistency (α > 0.71; Supplementary Table 1). The revised scale had excellent internal consistency, overall (α = 0.92) and for each of the six domains (0.72 < α < 0.83), as well as adequate item-total correlations (*r* ≥ 0.40; Supplementary Table 4). Test-retest reliability was assessed in a subsample of 40 participants, approximately 3 months later, according to Pearson’s *r* correlation coefficient, and found to be adequate (MHRM-20 total score: *r* = 0.89, *p* < 0.0001). Validity measures were also calculated and, as expected, were adequate (see below and **Table 2**). Because socio-demographic variables have been found to modulate the prognosis of schizophrenia (Westermeyer and Harrow, 1984; Wieselgren et al., 1996), discriminant validity was assessed across categories of age, education and disease duration, and found to be robust (Supplementary Table 5).

**Table 2:** Correlations between scores on the MHRM scale and other psychometric instruments.

	MHRM-20	
	*r*	*p*
TL30S-subjective	0.49	<0.0001
CAN user	-0.58	<0.0001
CAN staff	-0.48	<0.0001
GAF	0.65	<0.0001

*r, Pearson r correlation coefficient; p, p-value.*

### Relationship between CBR, SBR, Needs and Subjective QoL

As expected, the MHRM-20 score was positively correlated with QoL (TL30S-subjective) and GAF, and negatively correlated with CAN scores. In absolute terms, correlations for the total MHRM-20 scores were lowest with CAN-staff and highest with GAF (**Table 2**). We had similar findings when using the scores for the MHRM-30 scale (data not shown).

The association between MHRM-20 and GAF scores was further assessed in sequential linear regression models (**Tables 3** and **4**), with either MHRM-20 (**Table 3**, models 1–3) or GAF (**Table 4**, models 4–6) as the dependent variable. Base models (models 1 and 4) included only demographic (age, gender, marital status, and education) and clinical variables (substance abuse). The base models were first incremented with either GAF (model 2) or MHRM-20 (model 5), and then with TL-30S-subjective (models 3 and 6). In base models, demographic and clinical variables explained only 15% of the variance of the MHRM-20 score (model 1, *R*^2^ = 0.15), and 26% of the variance of the GAF score (model 4, *R*^2^ = 0.26). When GAF score was added to model 1, the resulting model had a 26% increment in explaining variance of the MHRM-20 score (model 2, *R*^2^ = 0.41). Similarly, when the MHRM-20 score was added to model 3, the resulting model had a 23% increment in explaining variance of the GAF score (model 5, *R*^2^ = 0.49). However, while the explanatory potential of the MHRM-20 model (model 2) was further incremented by 10% when the TL-30S-subjective score was included in the model (model 3, *R*^2^ = 0.51), when the TL-30S-subjective score was included in the GAF model (model 5), this had no impact (model 6, *R*^2^ = 0.49). Indeed, while the adjusted association between the MHRM-20 and TL-30S-subjective scores was statistically significant (β = 0.6 ± 0.2, *p* < 0.0001; model 3), that between the GAF and TL-30S-subjective score was not (β = -0.06 ± 0.2, *p* = 0.8; model 6).

**Table 3:** Sequential multiple linear regression models for the MHRM-20 score.

	Model 1 (*R*^2^ = 0.15)		Model 2 (*R*^2^ = 0.41)		Model 3 (*R*^2^ = 0.51)	
	β (SE)	*p*	β (SE)	*p*	β (SE)	*p*
Age	-0.2 (0.1)	0.1	0.09 (0.1)	0.5	0.06 (0.1)	0.6
Male gender	5.7 (3.8)	0.1	5.3 (3.2)	0.1	4.3 (2.9)	0.1
Marital status	8.1 (3.4)	0.02	-0.3 (3.1)	0.9	-0.9 (2.9)	0.8
Education	0.96 (0.5)	0.06	0.8 (0.4)	0.06	0.7 (0.4)	0.07
Substance abuse	-3.5 (3.4)	0.3	-3.2 (2.8)	0.3	-2.5 (2.6)	0.3
GAF			0.5 (0.08)	<0.0001	0.4 (0.07)	<0.0001
TL30S-subjective					0.6 (0.2)	<0.0001

*R^2^, R-squared statistic; β, Beta weight; SE, Standard error of β; p, p-value.*

**Table 4:** Sequential multiple linear regression models for the GAF score.

	Model 4 (*R*^2^ = 0.26)		Model 5 (*R*^2^ = 0.49)		Model 6 (*R*^2^ = 0.49)	
	β (SE)	*p*	β (SE)	*p*	β (SE)	*p*
Age	-0.6 (0.2)	<0.0001	-0.5 (0.1)	0.001	-0.5 (0.1)	0.001
Male gender	0.8 (4.3)	0.9	-2.8 (3.6)	0.4	-2.8 (3.6)	0.4
Marital status	16.6 (3.9)	<0.0001	11.5 (3.3)	0.001	11.5 (3.3)	0.001
Education	0.3 (0.6)	0.6	-0.3 (0.5)	0.5	-0.3 (0.5)	0.5
Substance abuse	-0.6 (3.8)	0.9	1.6 (3.2)	0.6	1.6 (3.2)	0.6
MHRM-revised			0.6 (0.1)	<0.0001	0.6 (0.1)	<0.0001
TL30S-subjective					-0.06 (0.2)	0.8

*R^2^, R-squared statistic; β, Beta weight; SE, Standard error of β; p, p-value.*

## Discussion

Here, we have contributed to clarify the construct of customer-based, subjective, or personal recovery (CBR), namely its relationship with another measure of recovery (service-based, objective, or clinical recovery – SBR) and an alternative measure of subjective experience (subjective QoL). Previous literature is not consensual regarding the dimensions within the complex construct of recovery. CBR is typically used to refer to personal experiential dimensions, including aspects such as empowerment and sense of hope (Schrank and Slade, 2007), and is frequently named subjective recovery. SBR is related to more clinical and social indicators, namely symptoms, functionality, participation in community and employment (Lloyd et al., 2010), and is generally equated to objective recovery. The use of such terminology has been misleading, with confusion between subjectivity and self-report resulting in constructs such as QoL to be considered as equivalent to CBR (Silverstein and Bellack, 2008; Lloyd et al., 2010; Roe et al., 2011). The results described here contribute to disambiguate these concepts. In fact, we found that the self-reported measure of CBR was correlated with two self-reported measures (subjective quality of life and a self-reported measure of needs – CAN-user), but was nevertheless better correlated to GAF, a clinician-rated measure of SBR. Linear regression models further confirmed the relationship between MHRM-20 and GAF, even when adjusting for demographic and clinical variables, while also showing that subjective QoL was related to MHRM-20 incrementally to GAF. The contrary, however, was not true, i.e., subjective QoL was not related to GAF incrementally to MHRM-20. Considered globally, these findings suggest that CBR, as evaluated by the MHRM-20 scale, could be used to measure global recovery, while also incorporating aspects of subjective QoL. Furthermore, these findings could contribute toward the reconciliation of clinical and consumer perspectives of recovery, as has been previously proposed (Davidson et al., 2005), while still demonstrating their complementary nature.

In previous empirical research, diverse findings have been reported relative to the relationship between CBR and SBR. Resnick et al. (2004) were the first to address this question. The authors found that, in a large sample of patients with schizophrenia, severity of symptoms, as measured by a shortened version of the Symptom Check List 90, was negatively associated to several dimensions of what was described as ‘recovery as an attitude or life orientation,’ namely life satisfaction, hope and optimism, knowledge about mental illness and services, and empowerment. While this data was used to argue against the polarization of recovery perspectives, it must be noted that these authors did not use CBR measures developed strictly from the accounts of users/patients. However, subsequent research using the two measures of CBR meeting this more stringent definition, i.e., the MHRM-30 and the recovery assessment scale (RAS) (Campbell-Orde et al., 2005; Andresen et al., 2010), have mostly confirmed a negative correlation between CBR scores and symptom-based measures of SBR, namely the Kessler-10 scale (Andresen et al., 2010), the Colorado Symptom Index (Stanhope et al., 2013) and the Scale for Assessment of Negative Symptoms and Scale for Assessment of Positive Symptoms (Norman et al., 2013). Others, using the Brief Psychiatric Rating Scale as a symptom-based measures of SBR, have not replicated this association (Roe et al., 2012), or have replicated it only when restricting analyses to subgroups of patients, according to age at disease onset (Roe et al., 2011).

Research comparing CBR and function-based measures of SBR or needs has been less consensual, motivating the work performed here. Lloyd et al. (2010) found a negative correlation between scores in the RAS and the CAN Short Appraisal Schedule. The strength of this correlation (*r* = -0.51) is similar to what, we found here between scores for the MHRM-20 and the CAN-user (*r* = -0.54) or the CAN-staff (*r* = -0.45). Andresen et al. (2010) found that MHRM-30 and GAF scores were positively correlated, but with a much weaker correlation than what we found (*r* = 0.24 vs. *r* = 0.64). Furthermore, in the latter and other studies (Andresen et al., 2010; Roe et al., 2011, 2012), an association between RAS and GAF scores was not found. However, as discussed by the respective authors (Roe et al., 2011), the patient populations where correlations between CBR and function-based SBR were weak or non-existent had particular characteristics. Specifically, patients were selected according to the presence of high support needs (Andresen et al., 2010), or were recruited at psychiatric rehabilitation residential centers (Roe et al., 2011, 2012). On the contrary, here and in the other study finding stronger correlations (Lloyd et al., 2010), patients were recruited from multiple clinical settings or a CMHS, and presumably were more representative of patients with SMI in general. Thus, we propose that our findings support prior research suggesting convergence between CBR and SBR (Andresen et al., 2010; Lloyd et al., 2010; Norman et al., 2013; Stanhope et al., 2013).

The convergence found, in this population, between CBR and function-based measures of SBR could be explained by different mechanisms. One possibility is that the distinction between CBR and function-based SBR is artificial, and that these two visions of recovery from SMI are actually reflections of a common and unitary construct. An alternate interpretation, however, is that CBR and SBR have common determinants, conditioning co-variation of their respective measures. One interesting possibility is that social factors, critical in the context of mental health (Fisher and Baum, 2010; Allen et al., 2014), determine aspects of both SBR and CBR. In fact, this possibility is partially supported by prior data, since there is evidence that social factors influence measures of CBR as well as GAF, even if to a lesser degree (Corrigan and Phelan, 2004; Hendryx et al., 2009). Several mechanisms are thought to underlie social influences on mental health, including social influence, self-esteem, sense of control, belonging, companionship, purpose and meaning, and perceived support availability (Thoits, 2011). Such proposed mechanisms are actually in line with many of the elements underlying the CBR construct, further supporting the connection between social factors and recovery. In fact, the hypothesis that common determinants, namely social factors, underlie the correlation between CBR and SBR, could explain why this finding is not common to all patient populations, as described above. In patients that have high support needs or reside at psychiatric rehabilitation centers (Andresen et al., 2010; Roe et al., 2011, 2012), it is conceivable that specificities of social characteristics, or interventions tailored to promote their modification, could modify the relationship between CBR and SBR. In any case, these hypotheses were not directly addressed with the work described here, and should be tested in the future.

The results of this study should be interpreted in the context of its cross-sectional experimental design. Thus, while the methods are adequate to query the relationship between the constructs of interest, they do not allow for exploration of causal relationships between them. Longitudinal studies are needed to explore the role of customer-based recovery recovery either as a prognostic factor or a measure of outcome. Furthermore, the use of the GAF score to assess functionality has been criticized in the past (Roy-Byrne et al., 1996). Nevertheless, the limitations of the GAF score are not consensual (Startup et al., 2002), and it is of frequent clinical use to assess functionality. Finally, comparisons of these results with those of previous studies are hindered by the fact that the patient sample described here has particular socio-demographic characteristics – patients are older, have low schooling and long disease duration. To the best of our knowledge, this is the first study on recovery from SMI conducted in a rural setting, which could explain such differences. However, the relationship between GAF and MHRM-20 does not seem to be dependent on the specificities of our sample, since it was mostly unchanged after adjustment for age, education level and duration of disease in linear regression models. Importantly, while the nature of the patient population in this study hinders the comparison with results from previous research, our findings also provide evidence, for the first time, that self reported measures of CBR can be used successfully in rural populations of patients with SMI, with low schooling and long disease duration.

## Conclusion

Our results demonstrate that, in certain patients populations, constructs for recovery from SMI are convergent, suggesting that recovery can be assessed using tools developed based on the experiences of users (CBR) as well as the knowledge of mental health experts (SBR). Specifically, we found that the MHRM-20, an instrument for self-assessment of CBR, assesses SBR and QoL, in addition to CBR, in chronic and elderly patients in a rural community mental health setting. Thus, when considering the ease of application and scoring of the MHRM-20 scale, relative to measures depending on user interview, such as the CAN scale, or on clinical evaluation, such as the GAF score, this measure gains appeal as an inexpensive tool for broad use in CMHSs, and possibly even in e-health instruments (Graffigna et al., 2014). Furthermore, given its underlying customer-based philosophy and development, the use of the MHRM-20, or similar measures, as tools for evaluation or assessment of outcome, holds promise to stimulate and further develop collaborative patient-clinician environments (Barello et al., 2012; Mullins et al., 2012; Domecq et al., 2014; Richards et al., 2015), firmly rooted in a recovery-oriented model for mental health services. Under a patient engagement framework, promoting transition from a more authoritative to a more collaborative model of healthcare provision (Graffigna et al., 2015), we expect that active monitoring of clinical outcomes using self-rated and patient-developed tools, such as the MHRM, will potentiate patient participation, involvement and empowerment, hopefully leading toward strengthening of the therapeutic alliance between patients and caregivers, and ultimately improving outcomes of clinical care (Barello et al., 2014; Graffigna et al., 2015).

## Author Contributions

AO-M, CM, and JG conceived and designed research. CM and MP acquired data. AO-M and MC analyzed data. AO-M, CM, MC, and JG interpreted data. AO-M and MC drafted the work that was critically revised by CM, MP, and JG. All authors approved the version to be published.

## Conflict of Interest Statement

AO-M and JG are recipients of an unrestricted research grant from Lundbeck Portugal. All the other authors declare that the research was conducted in the absence of any commercial or financial relationships that could be construed as a potential conflict of interest.
