# Supplementary material for: The Mental Health Recovery Measure Can Be Used to Assess Aspects of Both Customer-Based and Service-Based Recovery in the Context of Severe Mental Illness
Source: Front Psychol. 2016 Nov 3;7:1679. doi: 10.3389/fpsyg.2016.01679 (PMC5093119; doi:10.3389/fpsyg.2016.01679)
Supplement: Supplementary file 1 [file Table_1.pdf]

Supplementary Table 1. Reliability for MHRM total score and domain sub-scores.
Reliability was assessed for the original and revised MHRM scales using Cronbach's α (α). Domains with α greater than 0.7 are underlined.

	MHRM-original			MHRM-revised		
	**Construct**	**items**	**α**	**Construct**	**items**	**α**
**Total**	-	1-30	0.9	-	1, 2, 6-12, 14, 15, 17-24, 28	0.92
**Domains**	Overcoming stuckness	1-4	0.64	-		
	Self-empowerment	5-8	0.62	Empowerment	6, 7	0.79
	Learning and self-redefinition	9-12	0.81	Redefinition	1, 9, 10, 23	0.8
				Identity	11, 12	0.83
	Basic functioning	13-16	0.37	Social functioning	8, 14, 15, 28	0.81
	Overall well-being	17-20	0.71	Overall well-being	17, 19, 20	0.72
	New potentials	21-24	0.76	Optimism	2, 18, 21, 22, 24	0.82
	Advocacy/enrichment	27-30	0.44	-		
	Spirituality	25, 26	0.97	-		
