# Supplementary material for: The Mental Health Recovery Measure Can Be Used to Assess Aspects of Both Customer-Based and Service-Based Recovery in the Context of Severe Mental Illness
Source: Front Psychol. 2016 Nov 3;7:1679. doi: 10.3389/fpsyg.2016.01679 (PMC5093119; doi:10.3389/fpsyg.2016.01679)
Supplement: Supplementary file 2 [file Table_2.pdf]

Supplementary Table 2. Item-total correlations in the original MHRM scale.
Pearson's r correlation coefficient for each of the 30 items in the original MHRM scale, relative to the total score and each of the 8 domains. Items with item-total correlation relative to the total score less than 0.4 are underlined.

Domain/Item	Item-Total Correlation (r)	
	Domain	Total
A. Overcoming stuckness		
1. I work hard toward my mental health recovery.	0.44	0.51
2. Even though there are hard days, things are improving for me.	0.4	0.6
3. I ask for help when I am not feeling well.	0.35	0.39
4. I take risks to move forward with my recovery.	0.5	0.54
B. Self-empowerment		
5. I believe in myself.	0.34	0.48
6. I have control over my mental health problems.	0.45	0.46
7. I am in control of my life.	0.62	0.58
8. I socialize and make friends.	0.23	0.44
C. Learning and self-redefinition		
9. Every day is a new opportunity for learning.	0.59	0.68
10. I still grow and change in positive ways despite my mental health problems.	0.6	0.68
11. Even though I may still have problems, I value myself as a person of worth.	0.64	0.53
12. I understand myself and have a good sense of who I am.	0.67	0.55
D. Basic functioning		
13. I eat nutritious meals every day.	0.19	-0.03
14. I go out and participate in enjoyable activities every week.	0.27	0.43
15. I make the effort to get to know other people.	0.38	0.53
16. I am comfortable with my use of prescribed medications.	-0.01	0.06
F. Overall well-being		
17. I feel good about myself.	0.67	0.5
18. The way I think about things helps me to achieve my goals.	0.37	0.63
19. My life is pretty normal.	0.54	0.47
20. I feel at peace with myself.	0.41	0.43
G. New potentials		
21. I maintain a positive attitude for weeks at a time.	0.56	0.59
22. My quality of life will get better in the future.	0.49	0.52
23. Every day that I get up, I do something productive.	0.51	0.67
24. I am making progress towards my goals.	0.7	0.65
H. Spirituality		
25. When I am feeling low, my religious faith or spirituality helps me feel better.	0.95	0.19
26. My religious faith or spirituality supports my recovery.	0.95	0.23
I. Advocacy/enrichment		
27. I advocate for the rights of myself and others with mental health problems.	0.2	0.39
28. I engage in work or other activities that enrich myself and the world around me.	0.4	0.63
29. I cope effectively with stigma associated with having a mental health problem.	0.33	0.38
30. I have enough money to spend on extra things or activities that enrich my life.	0.08	-0.04
