# Supplementary material for: The Mental Health Recovery Measure Can Be Used to Assess Aspects of Both Customer-Based and Service-Based Recovery in the Context of Severe Mental Illness
Source: Front Psychol. 2016 Nov 3;7:1679. doi: 10.3389/fpsyg.2016.01679 (PMC5093119; doi:10.3389/fpsyg.2016.01679)
Supplement: Supplementary file 3 [file Table_3.pdf]

Supplementary Table 3. Rotated factor pattern of MHRM items.

22 MHRM items with item-total correlations relative to the total score greater than or equal to 0.4 were included in an exploratory factor analysis. Factorability of these 22 items was considered suitable since all items correlated 0.4 or more with at least one other item, the overall Kaiser-Meyer-Olkin measure of sampling adequacy was high (0.88), the Bartlett's test of sphericity was significant ($\chi^2 = 1015.6$, df = 231, p<0.0001), and the communality estimates were all above 0.3. Initial eigenvalues of the first 6 factors were above 1, while the remaining factors had eigenvalues below 1 and each explained less than 3% of variance. Thus, and also considering the scree plot of eigenvalues (not shown), a 6-factor solution was chosen. While both oblique (oblimin; not shown) and orthogonal (varimax) rotation methods were tested, these resulted in similar factors structures, and the varimax rotation was chosen as the definitive solution. Here we show varimax-rotated factors with the main factor loading for each item highlighted in bold. Considering the nature of the respective items, factors 1 to 6 were named, respectively, 'social functioning', 'redefinition', 'overall well-being', 'optimism', 'empowerment' and 'identity'. Item 24 ('I am making progress towards my goals') loaded similarly on two factors, but was considered conceptually closer to factor 4 than factor 1.

**Item**	**Rotated Factor Pattern**					
	**Factor1**	**Factor2**	**Factor3**	**Factor4**	**Factor5**	**Factor6**
8	**0.76**	0.06	-0.04	0.11	0.07	0.27
14	**0.73**	0.13	0.12	0.11	-0.05	0.1
15	**0.76**	0.15	-0.002	0.23	0.11	0.09
28	**0.46**	0.45	0.36	0.15	0.26	-0.21
1	0.25	**0.38**	0.04	0.25	0.24	0.16
4	0.19	**0.39**	0.25	0.12	0.24	0.03
5	0.01	**0.55**	0.11	0.17	0.11	0.3
9	0.18	**0.79**	0.16	0.3	0.04	0.21
10	0.24	**0.45**	0.08	0.4	0.3	0.27
23	0.38	**0.45**	0.32	0.13	0.32	-0.01
17	0.04	0.12	**0.74**	0.24	0.07	0.21
19	0.05	0.05	**0.69**	0.13	0.33	0.08
20	0.01	0.26	**0.45**	0.09	0.05	0.17
2	0.14	0.39	0.17	**0.4**	0.17	0.24
18	0.27	0.29	0.19	**0.65**	0.25	-0.02
21	0.15	0.19	0.44	**0.55**	0.14	0.03
22	0.19	0.21	0.17	**0.45**	0.14	0.13
24	0.38	0.33	0.31	**0.37**	0.14	0.12
6	0.03	0.12	0.17	0.26	**0.74**	0.05
7	0.08	0.26	0.24	0.13	**0.72**	0.3
11	0.29	0.21	0.16	0.06	0.1	**0.76**
12	0.18	0.23	0.31	0.14	0.2	**0.68**
**Factor Eigenvalues**	2.65	2.56	2.18	1.87	1.79	1.67
