# Supplementary material for: The Mental Health Recovery Measure Can Be Used to Assess Aspects of Both Customer-Based and Service-Based Recovery in the Context of Severe Mental Illness
Source: Front Psychol. 2016 Nov 3;7:1679. doi: 10.3389/fpsyg.2016.01679 (PMC5093119; doi:10.3389/fpsyg.2016.01679)
Supplement: Supplementary file 4 [file Table_4.pdf]

Supplementary Table 4. Item-total correlations in the revised MHRM scale.
Pearson's r correlation coefficient for each of the 20 items in the revised MHRM scale, relative to the total score and each of the 6 domains.

**Domain/Item**	**Correlation (r)**	
	**Domain**	**Total**
A. Empowerment		
6. I have control over my mental health problems.	0.65	0.49
7. I am in control of my life.	0.65	0.61
B. Redefinition		
1. I work hard toward my mental health recovery.	0.56	0.53
9. Every day is a new opportunity for learning.	0.66	0.67
10. I still grow and change in positive ways despite my mental health problems.	0.65	0.69
23. Every day that I get up, I do something productive.	0.59	0.65
C. Identity		
11. Even though I may still have problems, I value myself as a person of worth.	0.71	0.54
12. I understand myself and have a good sense of who I am.	0.71	0.6
D. Social functioning		
8. I socialize and make friends.	0.66	0.47
14. I go out and participate in enjoyable activities every week.	0.65	0.47
15. I make the effort to get to know other people.	0.73	0.54
28. I engage in work or other activities that enrich myself and the world around me.	0.47	0.62
E. Overall well-being		
17. I feel good about myself.	0.67	0.54
19. My life is pretty normal.	0.53	0.48
20. I feel at peace with myself.	0.43	0.4
F. Optimism		
2. Even though there are hard days, things are improving for me.	0.55	0.6
18. The way I think about things helps me to achieve my goals.	0.64	0.66
21. I maintain a positive attitude for weeks at a time.	0.63	0.61
22. My quality of life will get better in the future.	0.57	0.51
24. I am making progress towards my goals.	0.63	0.69
