# Supplementary material for: The Mental Health Recovery Measure Can Be Used to Assess Aspects of Both Customer-Based and Service-Based Recovery in the Context of Severe Mental Illness
Source: Front Psychol. 2016 Nov 3;7:1679. doi: 10.3389/fpsyg.2016.01679 (PMC5093119; doi:10.3389/fpsyg.2016.01679)
Supplement: Supplementary file 5 [file Table_5.pdf]

Supplementary Table 5. Discriminant validity summary.
MHRM scores were compared between age, education or disease duration categories, using unpaired t-tests – p-value (p) is reported.

**Age**	**Older than 50 years old** (n=46)	**50 years or younger** (n=49)	p
MHRM-original (mean ± SEM)	72.2 ± 13.7	77.2 ± 16.4	0.1
MHRM-revised (mean ± SEM)	46.4 ± 11.7	52.2 ± 12.6	0.02
**Education**	**Primary or less** (n=48)	**Higher than primary** (n=47)	p
MHRM-original (mean ± SEM)	71.2 ± 13.9	78.4 ± 15.8	0.02
MHRM-revised (mean ± SEM)	45.7 ± 11.7	53.1 ± 12.2	0.003
**Duration of disease**	**More than 20 years** (n=58)	**20 years or less** (n=37)	p
MHRM-original (mean ± SEM)	71.2 ± 13.6	80.5 ± 16.1	0.003
MHRM-revised (mean ± SEM)	46.2 ± 11.4	54.4 ± 12.5	0.001
